# Influence of transparency and opacity on the spelling of fricative phonemes

**DOI:** 10.1590/2317-1782/20232021212en

**Published:** 2023-06-02

**Authors:** Larissa Paschoal, Lourenço Chacon

**Affiliations:** 1 Programa de Pós-graduação em Estudos Linguísticos, Universidade Estadual Paulista - UNESP - São José do Rio Preto (SP), Brasil.

**Keywords:** Handwriting, Language, Phonetics, Child, Speech Language and Hearing Sciences

## Abstract

**Purpose:**

(1) to verify to what extent the occurrence of possible errors is influenced by the relationship (opaque/transparent) between fricative phonemes and the graphemes with which they can be spelled; (2) verify the differences (if present or not) of relationship types among the phonemes that present common graphemic relationships.

**Methods:**

We analyzed 750 textual productions from children in the first year of Elementary School (ES), and conducted a survey of the frequency of correct answers and errors in all fricative phonemes of Brazilian Portuguese (BP).

**Results:**

The errors occurred in greater numbers in the group of phonemes with opaque spelling when compared with the number of errors in the group of phonemes with transparent spelling. In the first group, the errors showed a non-symmetrical behavior, since they varied according to the possibilities of graphemes for each phoneme. In the second group, the errors showed a symmetrical behavior.

**Conclusion:**

Given the symmetry in the errors of the phonemes of the first group and the non-symmetry of those of the second group, our results point to a gradation in the occurrence of errors, which varies as a function of the transparency and degree of opacity in the relations between phonemes and graphemes of a same class.

## INTRODUCTION

The writing system of Brazilian Portuguese (BP) is based on the alphabetic principle, that is, on the principle that graphic segments (graphemes) correspond to phonic segments (phonemes)^(1)^. In an ideal alphabetic writing system, a grapheme would correspond to only one phoneme and, conversely, a phoneme would correspond to only one grapheme. However, we observed both regularities and irregularities in this correspondence in BP. Regularities can be defined through transparency relations - in which a phoneme corresponds to only one grapheme and vice versa. Irregularities, in turn, can be defined through opacity relationships - in which a phoneme corresponds to more than one grapheme, or the opposite, a grapheme corresponds to more than one phoneme^(2)^.

Although BP has a transparent writing system, motivated by phonological principles, a considerable number of opaque relationships can be observed in it. Thus, spelling words according to traditional spelling can be challenging during literacy and, therefore, susceptible to the occurrence of unconventional records (so-called spelling errors). Understanding the nature of the principles and processes that can influence, facilitate and/or hinder children's spelling, therefore, has been the subject of investigations in different areas of knowledge from different perspectives.

These investigations show the concern to characterize the performance of children in skills considered as metalinguistic, especially those involved in the so-called phonological awareness (PA) and in other factors associated with them, such as, for example, the vocabulary and intelligence^(3,4)^; as well as verifying to what extent the PA would help the learning of reading and writing^(5)^. Some investigations sought to characterize and evaluate the performance of children with Attention Deficit Hyperactivity Disorder (ADHD) and dyslexia, since, according to this literature, such skills could help the diagnosis and, when used in interventions, favoring the learning of reading/writing, although neurobiological factors may influence this performance^(6,7)^.

The literature also shows investigations that aimed to verify the relationship between the performance in PA activities and the occurrence of spelling errors in children with typical and atypical phonological development^(8)^. There are also investigations that, in addition to the PA, sought to verify the isolated influence of the so-called morphological awareness in the spelling of Elementary School (ES) children^(9)^.

Finally, in these investigations, there is a concern to verify the effects, both of opacity and orthographic transparency, in the so-called acquisition of reading in different alphabetic-based writing systems^(10-12)^. Finally, some investigations also sought to understand the effects of orthographic opacity in the writing of bilingual children^(13)^.

Although these investigations report an overview of (i) the possible zones that would facilitate and/or hinder the learning of orthography and (ii) the role of skills considered as metalinguistic in literacy, few references to phonetic-phonological aspects of the language were observed - an absence that calls attention, given the important role of these aspects in the constitution of alphabetic writing systems.

Especially because some reports aimed to understand more detailed aspects related to orthography in the literacy period, involved in the relationship between orthographic characteristics and phonetic-phonological aspects of the language. These reports show concern to understand links between syllable structure and orthography since the position and/or syllabic complexity could interfere with the conventional orthographic record^(14-16)^. Some investigations sought to verify the interference of perception and auditory training in the reduction of phonologically based orthographic errors observed in children with temporal auditory processing disorder^(17)^.

In addition to these, there are investigations based on more systematic relationships between phonology and orthography, in which the analysis of spelling errors is observed as a revealing source of children's knowledge about the phonology of the language and reading and writing practices^(18)^, as well as the children's orthographic performance regarding the registration of phonemes of specific phonological classes^(19-22)^. These latest investigations indicate how the phoneme/grapheme relationship is registered as a function of the consonant phonological classes of BP. There are also indications of conflict zones in this relationship. However, these works were not concerned with the difference in the establishment of the phoneme/grapheme relationship within the same phonological class.

Thus, this present study aimed to investigate to what extent would the records of graphemes that compose the same phonological class present points of symmetry/non-symmetry in the orthographic register. We hypothesize that the orthographic record of phonemes of the same class would be influenced by the type of phoneme/grapheme relationship it presents. Thus, correspondences in which transparency relations are observed would be less conflicting and, therefore, less susceptible to errors than the spelling of phonemes in which opacity relations are observed. Based on this hypothesis, our main goals are (1) to verify to what extent the occurrence of possible errors is influenced by the relationship (opaque/transparent) between fricative phonemes and the graphemes with which they can be spelled; (2) verify the differences (if present or not) of relationship types among the phonemes that present common graphemic relationships.

The choice to investigate the orthography of fricative phonemes comes from the complexity observed in the phoneme/grapheme correspondence observed in this class. As previously mentioned, if we consider the position of simple syllabic attack, although the phoneme/grapheme correspondence predominates in BP motivated by transparent relationships - of the nineteen consonant phonemes, twelve present regular correspondence in this syllabic position^(23)^ -, it is also found in it a considerable number of correspondences motivated by opaque relationships (seven of the nineteen consonant phonemes). The largest number of opacity relations in these correspondences is precisely in the class of fricative phonemes, since four (/s/, /z/, /ʃ/, /ʒ/) of the six phonemes that make up this class in BP have opaque writing, that is, irregular.

## METHODS

We used a set of textual productions taken from a database that integrates the project *Links between characteristics of oral and literate practices in the acquisition and development of writing* (CNPq - Process 400183/2009-9), approved by the FFC/UNESP Research Ethics Committee under number 0138/2010.

The database comprises 2,972 textual productions by 300 children who attended Elementary School I, between 2001 and 2004, in two Municipal Elementary Schools in São José do Rio Preto, State of São Paulo. Only the texts of children who had authorization from their parents or guardians to participate in the task by signing the Informed Consent Term were included^^*^^ .

The textual productions were collected by researchers from *Grupo de Pesquisa ‘Estudos sobre a Linguagem’* (GPEL/CNPq). Approximately every fifteen days, these researchers held pedagogical workshops that resulted in different thematic proposals for writing. Then, according to the theme presented, the children were asked to produce a text. These proposals mobilized the production of texts of different discursive genres by the children. It should be noted that the proposals followed the pedagogical planning of the schools. Thus, it is not a question of setting up a database with an experimental character, but with a character closer to the children's school routine.

For the investigation reported in this article, from the bank material, the textual productions of all children who attended the first grade of Elementary School in 2001 in the two schools were selected, namely: 75 children. None of them had any complaints about learning to write, nor about language development in their speech. These are children with typical development of both speech and writing. Such information was collected from their parents and the schools' pedagogical team.

The productions refer to 14 thematic proposals presented throughout the school year. Chart 1 shows the themes of each of these proposals, as well as the date of presentation and collection.

**Chart 1:** Thematic proposals

**Proposal**	**Theme**
1	Prior knowledge about hearing
2	Lecture report on hearing
3	Letter to Renata 01
4	Letter to Renata 02
5	The Country Mouse and the City Mouse
6	The True Story of the Three Little Pigs: Diary of a Wolf
7	In need of glasses?
8	Dengue
9	Shopping list 01
10	Shopping list 02
11	Cake recipe
12	Prior voice survey
13	Talk about the voice
14	Christmas card

Source: Research database.

A total of 1,050 textual productions (75 children x 14 thematic proposals) were expected for analysis. However, due to absences and the impossibility of interpreting some records, 750 productions were analyzed, and 300 productions were discarded.

Among the 750 productions analyzed, all occurrences of graphemes that referred to fricative phonemes of Brazilian Portuguese (BP) in the syllabic position of simple attack were verified. These occurrences were classified as: CORRECT ANSWERS, when the spelling was in accordance with orthographic conventions; and ERRORS, when the spelling was at odds with conventional spelling. Correct answers and errors were counted in all target fricative phonemes, /f/, /v/, /s/, /z/, /ʃ/ and /ʒ/, to determine the frequency of appearance of each one of these phonemes in the sample.

In other words, for each target phoneme, the total of possibilities of occurrences in each text of the entire analyzed sample was first raised. Of this total, the number of correct answers and errors was verified. Still for each target phoneme, this number was transformed into percentage numbers and relative numbers. For example, the phoneme /f/ had a total of 1604 recording possibilities in the entire sample. Of these 1604 possibilities: 1562 corresponded to correct answers; 52 to errors. In percentage terms: 1604 possibilities = 100%; 1562 correct answers = 97%; 52 errors = 3%. To arrive at the relative numbers, the percentage of correct answers (97%) and errors (3%) divided by one hundred was considered. Thus, the relative numbers of 0.97 and 0.03 were reached, respectively. Considering that the objectives of the investigation reported here concern, primarily, spelling errors, in the statistical analysis only the relative numbers of these errors were used.

This procedure was necessary since the frequency of appearance of each of these phonemes, in an uncontrolled sample, does not occur similarly. Therefore, the need to use a relative number for errors, that is, a value that ranged from 0 to 1 for the purpose of statistical analysis.

The errors, in turn, were grouped (according to the type of phoneme/grapheme relation) into errors in transparent relations /f/, /v/; and, in errors in opaque relationships /s/, /z/, /ʃ/, /ʒ/. This grouping allowed, according to the first aim, to verify whether the total occurrence of errors would be influenced by the type of relationship (transparent/opaque) that the phonemes maintain with the graphemes. Also, according to the second aim, the grouping was conducted in order to verify, between these two types of relationships, if the phonemes that present common graphemic relationships presented, or not, differences between them.

We used the STATISTICA software, version 7.0. to perform descriptive and inferential analyzes. For the descriptive analysis, we used a measure of central tendency (mean) and a measure of dispersion (standard deviation). For the inferential analysis, a significance level of α ≤ 0.05 and a confidence interval of 95% were established. Nonparametric tests ANOVA and Kendall Coefficient of Concordance were applied for the analysis of multiple simple dependent variables; and the Wilcoxon Matched and Pairs test to compare two simple variables and for post hoc analysis. In all analyses, we used the values corresponding to the relative measures.

## RESULTS

The study aimed to understands to what extent the errors would depend on the relations of transparency or opacity that the fricative phonemes maintain with the graphemes with which they can be spelled. To this end, the 1,491 occurrences of records classified as errors in the total sample were arranged into two groups, whose results are shown in Table 1:

**Table 1:** Comparison between errors based on the type of phoneme/grapheme relationship

P/G Ratio	Number of occurrences	Average	Standard Deviation	Inferential Analysis
Transparent	*112*	0.78	0.13	T = 3.0
				Z= 7.3
Opaque	*1379*	0.89	0.19	p = 0.00 (*)

Source: Research data.

**Caption:** P/G - Phoneme/Grapheme;

(*) indicates statistical difference. Test: Wilcoxon Matched Pairs.

Table 1 shows that 112 errors (7.51%) occurred in phonemes whose spelling is transparent and 1,379 (92.49%) in those with opaque spelling, a distribution that proved to be statistically significant.

As proposed in the second objective, we observed, among the two types of relationships (opaque/transparent), if the phonemes that present common graphemic relationships showed differences between them. The results regarding the comparison between the phonemes that present a transparent graphemic relationship are shown in Table 2:

**Table 2:** Comparison between spelling errors of phonemes with transparent spelling

**Target phoneme**	**Number of occurrences**	**Average**	**Standard Deviation**	**Inferential Analysis**
/f/	52	0.22	0.38	T = 263.50
				Z= 0.58
/v/	60	0.26	0.40	p = 0.56

Source: Research data. Wilcoxon Matched Pairs Test.

As for the comparison between the phonemes with a transparent phoneme/grapheme relationship, shown in Table 2, it appears that the number of occurrences of errors in the phonemes /f/ and /v/ is remarkably close, 52 (46.43%) and 60 (53.57%), respectively. With similar mean and standard deviation values. In the inferential analysis, no statistically significant difference was observed in the distribution of errors.

Still, regarding the second objective of this investigation, Table 3 shows the results of the comparison between the phonemes that have an opaque relationship with the graphemes by which they can be spelled:

**Table 3:** Comparison between spelling errors of phonemes with opaque spelling

**Target phoneme**	**Number of occurrences**	**Average**	**Standard Deviation**	**Sum of ranks**	**Inferential Analysis**
/s/	920	0.64	0.23	281^1,2,3^	Chi- sqr. 127.92
/z/	188	0.15	0.17	170^1,4^	df = 3
/ʃ/	193	0.13	0.13	169^2,5^	p= 0.00*
/ʒ/	78	0.05	0.07	120^3,4,5^	

Source: Research data. Friedman ANOVA and Kendall Coeff test of Concordance.

**Caption:** (*) indicates a statistically significant difference. Post-hoc analysis: Wilcoxon Matched Pairs test, equal superscript numbers indicate statistically significant difference.

Regarding the comparison between the phonemes with an opaque phoneme/grapheme relationship, we can observe: (i) greater occurrence of errors in the spelling of the phoneme /s/ (920 - 66.71%); (ii) occurrence in absolute values and similar percentages between the phonemes /z/ and /ʃ/, in the order, 188 (13.63%) and 193 (13.99%); and (iii) lower number of occurrences in the spelling of the phoneme /ʒ/, 78 (5.67%). As for the inferential analysis, a statistically significant difference was observed in the comparison between the phonemes of this category. In the post hoc analysis, we found that the phoneme /s/ differs from all other phonemes; the phonemes /v/ and /ʃ/, in turn, showed differences only with the phonemes /s/ and /ʒ/; and, finally, the phoneme /ʒ/ presented differences with the phonemes /s/, /z/ and /ʃ/.

## DISCUSSION

Regarding the tendency found for the first objective, the occurrence of errors was shown to be dependent on the type of relationship that phonemes maintain with graphemes. That is, we observed that phonemes that present a transparent relationship with the graphemes with which they can be spelled represented a small percentage (7.51%) of the occurrence of errors when compared to phonemes that present an opaque graphemic relationship. The latter corresponded to most of the sample (92.49%).

The main explanatory hypothesis for this tendency is the number of graphemic possibilities that the phonemes present. In fact, in relations of transparency, a phoneme has only one possibility of registration; in opacity relations, in turn, a phoneme can present two or more possibilities of registration; therefore, the probability of errors occurring increases.

There is, therefore, in this first trend, an important aspect to be considered in the occurrence of spelling errors: the type of relationship that the phoneme maintains with the grapheme by which it can be spelled. Although aimed at reading and developed with a theoretical basis that differs from that of the present article, two studies observe evidence of areas of reading decoding facilities in phoneme/grapheme correspondences with transparent orthography^(10,11)^. Also, in work developed with another type of theoretical-methodological framework and focused on global characteristics of writing (and not just spelling), areas of difficulty in correspondences with opaque orthography were highlighted^(12)^.

Still, in relation to the first trend, we found investigations also based on phonetic-phonological aspects of the language that raised important aspects that can interfere with the occurrence of orthographic errors, such as: (i) phonological classes^(21)^ and (ii) syllabic complexity^(16)^_._

As for the trends found for the second objective, firstly, no difference was observed between the phonemes that present transparent spelling (/f/ and /v/) in the occurrence of errors. This result suggests that these phonemes work very similarly in spelling, possibly because they present a single orthographic possibility, thus reducing the probability of being spelled with errors. This trend confirms that verified with data from writing systems that present a high level of regularities between phonemes and graphemes, such as Italian, Finnish and Indonesian, in which strong reflections of phonology in orthography are observed. These regularities reduce the possibility of errors occurring in reading and writing tasks when compared to the occurrence of errors in writing systems with a high degree of opacity, such as English, for example^(25)^.

The second trend found showed non-symmetry between the occurrence of errors in the spelling of phonemes that present an opaque relationship with their corresponding graphemes (/s/, /z/, /ʃ/ and /ʒ/). Thus, despite maintaining the same type of relationship, these phonemes do not seem to work similarly in spelling. A possible explanatory hypothesis for this tendency is the presence of multiple spelling possibilities that the same phoneme admits. In the fricative phonemes, their orthographic register can present from two to nine graphemic possibilities in the syllabic position of simple attack. In addition to this quantitative aspect, among these possibilities, the following can operate (a) syllabic position rules - for example, when registered between vowels, the grapheme {s} refers to the phoneme /z/; this same grapheme, when registered at the beginning of a word (*santa*), or after graphemes that refer to phonemes of the liquid class (*aniversário*) and nasal class (*mansão*), starts to refer to the phoneme /s/; (b) occurrence of digraphs - situation in which the phoneme presents as a graphemic possibility the combination of two elements, for example, the letters 'c' and 'h', when together 'ch' start to refer to the phoneme /ʃ/; and (c) competition relations - when two or more graphemes can be used in the same position and the same syllabic context (*“música”; “zebra”; “exame”*, situation in which all graphemes refer to the phoneme /z/)^(1)^.

As for the non-symmetry between the fricative phonemes that present opaque orthography, we observed (i) greater occurrence of errors in the spelling of the phoneme /s/; (ii) similar behavior between errors in the phonemes /z/ and /ʃ/; (iii) non-relationship between errors in the spelling of the phoneme /ʒ/ with the other fricative phonemes. These trends suggest that, in addition to presenting divergences, they also present a gradation.

Thus, the greater occurrence of errors in the spelling of the phoneme /s/ can be explained by the great opacity they maintain, since, in the syllabic position of simple attack, this phoneme has nine possibilities of being spelled {s, ss, c, ç, sc, sç, xs, xc, x}, being the most opaque phoneme when compared to all the others in BP (both in the fricative class and in the other classes: stops, nasals, liquids and vowels). In addition to this large number of orthographic possibilities, other complexities in its orthography are observed, such as: (a) the possibility of digraphs - for example, the writing of the words {*a* ***ss*** *ar*} {*e* ***xc*** *eção*}; {*n* ***as*** *cer*}; (b) competition relations - as in the words {***s*** *ela*}, {***c*** *eia*}, {*pa* ***ss*** *eio*}, {*a* ***c*** *eite*} in which all graphemes refer to the phoneme /s/; and (c) reduced number of contextual rules that provide for the use of one or another grapheme, requiring, in most cases, memorization. Thus, spelling this phoneme according to orthographic conventions can be a difficult task for children in early grades. A similar result was observed in the analysis of spelling errors in the early grades, in which the spelling involving the phoneme /s/ corresponded to most of the errors found^(18)^.

The similar behavior observed in the occurrence of errors in the spelling of the phonemes /z/ and /ʃ/, in turn, may be associated with two characteristics of the spelling of these phonemes: reduced number of graphemic possibilities and only one possibility of digraph. The orthographic record of the phoneme /z/ admits three graphemic possibilities {z}, {s}, {x}, without any possibility of digraph; on the other hand, the orthographic record of the phoneme /ʃ/, although it admits only two possibilities of registration, {x}, {ch}, one of them is using a digraph. Still, none of these two phonemes presents a rule that determines the use of their graphemes according to the orthography, since “when more than one letter can, in the same position, represent the same sound, the option for the correct letter in a word is, in purely phonological terms, entirely arbitrary.”^(1:21)^.

In the third trend found among the errors that involved an opaque phoneme/grapheme relationship, we found that the phoneme /ʒ/ was not related to any of the other three phonemes with opaque writing /s/, /z/, /ʃ/. When compared to the other fricative phonemes, the phoneme /ʒ/ presents only two possibilities of graphic register {j} and {g}. Among these two possibilities, one can be predicted by contextual rules since the grapheme {g} only corresponds to the phoneme /ʒ/ when preceded by the vowels /e/ and /i/. In addition, the spelling of this phoneme does not admit the possibility of digraphs, further reducing the complexity of spelling it. Thus, considering this result, we inferred that this phoneme behaves orthographically in an intermediate gradation between the phonemes with transparent writing and the phonemes with opaque writing.

Considering all the trends found regarding the characteristics of the relationship between phonetic-phonological aspects and orthographic aspects of fricative phonemes, there are indications that, although these phonemes work similarly in speech, the same does not occur in the orthographic aspect of writing. In speech, these phonemes usually work in pairs that are differentiated by a minimal unit, which, in this case, is the presence or absence of the feature of voicing feature. Thus, in the speech of children with typical and atypical phonological development, repair strategies that involve devoicing may occur - for example, the phoneme /v/, when produced without vocal fold vibration, corresponds to the phoneme /f/^(26)^. However, we observed that, in orthography, other factors can influence the registration of these phonemes, such as the number of graphemic possibilities, the occurrence of digraphs and the presence/absence of rules that define the use of a grapheme according to with the current spelling standard.

Finally, regarding the trends found, although BP has a transparent writing system and, according to the literature, systems with regular writing are less susceptible to errors on the part of children in literacy^(25)^, we observed the importance of specifically analyzing the phonemes that make up the same phonological class. In addition, it is important to characterize how the relationships that children considered typical establish between phonemes and graphemes develop and what are their conflict zones, since, in the literature, the occurrence of reading errors and writing, especially in its orthographic aspect, are used as diagnostic criteria for learning disorders^(6,7,25)^.

The results found in this research contribute to a better understanding of the possible spelling facilities and/or difficulties in the initial series of literacy, on the part of professionals in the fields of health and education. Especially because the results point to the importance of these professionals having knowledge about the relationships, sometimes quite complex, between phonetic-phonological aspects of the language and aspects of the conventions that regulate orthography, so that this knowledge provides subsidies for clinical and educational practices with children's writing.

The results and trends found direct further investigations that aim to characterize the nature of orthographic errors in different phonological classes, in other syllabic contexts and, also, in the sequence of years that make up the Literacy Cycle. Thus, the present study has limitations.

## CONCLUSION

Our results allowed to verify that the phonemes in which there are transparent relationships with their corresponding graphemes were much less susceptible to the occurrence of errors than the phonemes that maintain opaque relationships with their corresponding graphemes. These results partially corroborate the initial hypothesis that the orthographic record of the same class would be influenced by the type of phoneme/grapheme relationship it presents.

However, differently from what was predicted in the hypothesis, among the opaque ones, factors such as: (a) the number of graphemic possibilities; (b) competition between graphemes, that is, the absence or limited number of rules that define orthographically appropriate usage; and (c) presence/absence of digraphs, interfered in the occurrence of errors.

We verified points of symmetry between phonemes with transparent orthography, since the errors occurred in a similar way and smaller numbers; and non-symmetry points among the phonemes that present opaque orthography. Given this symmetry and asymmetry in the errors, the results obtained point to a gradation in their occurrence, which varies according to the transparency and degree of opacity in the relationships between phonemes and graphemes of the same class. This gradation can be divided into four groups, considering the following order from the most transparent to the opaquest: 1st /f/ and /v/; 2nd /ʒ/; 3rd /z/ and /ʃ/; and 4th /s/.

## Footnotes

* Although, according to the resolution of the National Health Council of Brazil - CNS No. 466/12^24^ , the authorization of both the parents and the participating children is mandatory, we emphasize that the collection took place in a period prior to this resolution, in which only the assent of the parents and/or guardians allowed data collection.

## References

[1] Lemle M (2009). Guia teórico do alfabetizador..

[2] Veloso J. (2005). Algumas considerações gerais sobre transparência e opacidade fonêmica na escrita do português e outras questões. Da investigação às práticas. Estudos de Natureza Educacional.

[3] Schafer CM, Quitaiski LF, Giacchini V (2017). Desempenho em consciência fonológica e erros de escrita de crianças submetidas a diferentes métodos de alfabetização. Rev Distúrb Comun.

[4] Leite RCD, Brito LRM, Martins-Reis VO, Pinheiro AMV (2018). Consciência fonológica e fatores associados em crianças no início de alfabetização. Rev Psicopedagogia..

[5] Guaresi R, Oliveira J, Oliveira E, Teixeira L (2017). A consciência fonológica e o vocabulário no aprendizado da leitura e da escrita na alfabetização. Rev (Con) Textos Ling..

[6] Gonçalves-Guedim TF, Capelatto IV, Salgado-Azoni CA, Ciasca SM, Crenitte PAP (2017). Desempenho do processamento fonológico, leitura e escrita em escolares com transtorno de déficit de atenção e hiperatividade. Rev CEFAC.

[7] Arfé B, Vardanega T, Montuori C, Lavanga M (2019). Coding in primary grades boosts children’s executive functions. Front Psychol.

[8] Donicht G, Ceron MI, Keske-Soares M (2019). Erros ortográficos e habilidades de consciência fonológica em crianças com desenvolvimento fonológico típico e atípico. Rev CoDAS.

[9] Guimarães SB, Mota MMPE (2018). Consciência morfológica e ortografia. Uma relação para além da consciência fonológica?. Estud Pesqui Psicol.

[10] Rakhlin NV, Mourgues C, Cardoso-Martins C, Kornev AN, Grigorenko EL. (2019). Orthographic processing is a key predictor of reading fluency in good and poor readers in a transparent orthography. Contemp Educ Psychol.

[11] Milankov V, Golubović S, Krstić T, Golubović S (2021). Phonological awareness as the foundation of reading acquisition in students reading in transparent orthography. Int J Environ Res Public Health.

[12] Calero A, Calero-Perez E Opacidad ortográfica y aprendizaje de la comprensión lectora en español. Ocnos.

[13] Kaani B (2021). Writing proficiency across diverse writing systems. Zambia Interdisc J Educ..

[14] Sucena A (2017). Sensibilidade precoce às combinações ortográficas entre crianças falantes do Português Europeu. Investig Prat.

[15] Pachalski L, Miranda ARM (2018). A metátese na aquisição da escrita: simetrias e assimetrias entre fonologia e ortografia. Filol Linguíst..

[16] Miranda ARM (2019). As sílabas complexas: fonologia e aquisição da linguagem oral e escrita. Fórum Linguistic..

[17] Pires MM, Schochat E (2019). The effectiveness of an auditory temporal training program in children who present voiceless/voiced-based orthographic errors. PLoS One.

[18] Miranda ARM (2020). Um estudo sobre a natureza dos erros (orto)gráficos produzidos por crianças dos anos iniciais. Educ Rev.

[19] Vaz S, Pezarini IO, Paschoal L, Chacon L (2015). Características da aquisição da ortografia de consoantes soantes em crianças de um município paulista. Rev CoDAS.

[20] Pezarini IO, Vaz S, Paschoal L, Chacon L.  (2015). Relações entre aspectos ortográficos e fonético-fonológicos de fonemas oclusivos. Rev CEFAC.

[21] Chacon L, Paschoal L, Vaz S, Pezarini IO (2016). Classes fonológicas e ortografia infantil. Rev do GELNE..

[22] Vaz S, Chacon L (2020). Coocorrência de traços fonológicos em substituições ortográficas de fonemas soantes. Rev CoDAS.

[23] Camara JM (1970). Estrutura da língua portuguesa.

[24] Brasil (2012). Resolução nº 466, de 12 de dezembro de 2012: diretrizes e normas regulamentadoras de pesquisa envolvendo seres humanos.

[25] Borleffs E, Maasen BAM, Lyytinen H, Zwarts F Cracking the code: the impact of orthographic transparency and morphological-syllabic complexity on reading and developmental dyslexia. Front Psychol.

[26] Ceron MI, Gubiani MB, Oliveira CR, Gubiani MB, Keske-Soares M. (2017). Ocorrência do desvio fonológico e de processos fonológicos em aquisição fonológica típica e atípica. Rev CoDAS.

