# Supplementary material for: Influence of transparency and opacity on the spelling of fricative phonemes
Source: Codas. 2023 Jun 2;35(3):e20210212. doi: 10.1590/2317-1782/20232021212en (PMC10266796; doi:10.1590/2317-1782/20232021212en)
Supplement: Translation

# Translation: Influência da transparência e da opacidade na ortografia de fonemas fricativos.

**DOI:** 10.1590/2317-1782/20232021212pt

**Authors:** Larissa Paschoal, Lourenço Chacon

**Affiliations:** 1 Programa de Pós-graduação em Estudos Linguísticos, Universidade Estadual Paulista - UNESP - São José do Rio Preto (SP), Brasil.

**Keywords:** Escrita Manual, Linguagem, Fonética, Criança, Fonoaudiologia

## Abstract

**Objetivo:**

(1) verificar em que medida a ocorrência de possíveis erros seria influenciada pelo tipo de relação (opaca/transparente) que os fonemas fricativos mantêm com os grafemas com que podem ser ortografados; (2) verificar, dentre esses dois tipos de relações, se os fonemas que apresentam relações grafêmicas comuns apresentam ou não diferenças entre si.

**Método:**

Foram analisadas 750 produções textuais de crianças do 1º ano do Ensino Fundamental (EF). Nessas produções, foi realizado o levantamento da frequência de acertos e de erros na ortografia de fonemas fricativos do Português Brasileiro (PB).

**Resultados:**

Os erros ocorreram em maior número no grupo de fonemas com ortografia opaca quando comparados com o número de erros no grupo de fonemas com ortografia transparente. No primeiro grupo, os erros apresentaram comportamento não simétrico, já que variaram em função das possibilidades de grafemas para cada fonema. Já no segundo grupo, os erros apresentaram comportamento simétrico.

**Conclusão:**

Dadas a simetria nos erros dos fonemas do primeiro grupo e a não simetria naqueles do segundo grupo, os resultados obtidos apontam para uma gradação na ocorrência de erros, que varia em função da transparência e do grau de opacidade nas relações entre fonemas e grafemas de uma mesma classe.

## INTRODUÇÃO

O sistema de escrita do Português Brasileiro (PB) é baseado no princípio alfabético, ou seja, no princípio de que segmentos gráficos (grafemas) correspondem a segmentos fônicos (fonemas)^(1)^. Em um sistema de escrita alfabética ideal, um grafema corresponderia a somente um fonema e, inversamente, um fonema corresponderia a apenas um grafema. Todavia, observamos no PB tanto regularidades, quanto irregularidades nessa correspondência. As regularidades podem ser definidas por meio de relações de transparência - em que um fonema corresponde a apenas um grafema e vice-versa. As irregularidades, por sua vez, podem ser definidas por meio de relações de opacidade - em que um fonema corresponde a mais de um grafema, ou o contrário, um grafema corresponde a mais de um fonema^(2)^.

Embora o PB seja um sistema de escrita predominantemente transparente, motivado, sobretudo, por princípios fonológicos, observa-se nele considerável número de relações opacas. Dessa forma, grafar as palavras de acordo com a ortografia tradicional pode ser desafiador durante a alfabetização e, logo, suscetível à ocorrência de registros não-convencionais (os chamados erros ortográficos). Compreender a natureza dos princípios e dos processos que podem influenciar, facilitar e/ou dificultar a ortografia de crianças, portanto, vem sendo alvo de investigações em diferentes áreas de conhecimento sob diferentes perspectivas.

Nessas investigações, observa-se, por um lado, a preocupação de se caracterizar o desempenho de crianças em habilidades consideradas como metalinguísticas, sobretudo naquelas envolvidas na chamada consciência fonológica (CF) e em outros fatores associados a elas, como, por exemplo, o vocabulário e a inteligência^(3,4)^; bem como verificar em que medida a CF auxiliaria o aprendizado da leitura e da escrita^(5)^. Observam-se, também, investigações que buscaram caracterizar e avaliar o desempenho de crianças com Transtornos do Déficit de Atenção e Hiperatividade (TDAH) e com dislexia, uma vez que, de acordo com essa literatura, tais habilidades poderiam auxiliar o diagnóstico e, quando utilizadas em intervenções, favorecer o aprendizado da leitura/escrita, embora fatores neurobiológicos possam influenciar esse desempenho^(6,7)^.

Na literatura, observam-se, também, investigações cujo objetivo foi verificar a relação entre o desempenho em atividades de CF e a ocorrência de erros ortográficos em crianças com desenvolvimento fonológico típico e atípico^(8)^. Observam-se, ainda, investigações que, para além da CF, buscaram verificar a influência isolada da chamada consciência morfológica na ortografia de crianças do Ensino Fundamental (EF)^(9)^.

Nessas investigações, observa-se, por fim, a preocupação de verificar os efeitos, tanto da opacidade, quanto da transparência ortográfica, na chamada aquisição da leitura em diferentes sistemas de escrita com base alfabética^(10-12)^. Observam-se, por fim, investigações que também buscaram compreender os efeitos da opacidade ortográfica na escrita de crianças bilíngues^(13)^.

Embora tenha sido observado, nessas investigações, um panorama geral (i) das possíveis zonas que facilitariam e/ou dificultariam o aprendizado da ortografia e (ii) do papel das habilidades consideradas como metalinguísticas na alfabetização, poucas referências a aspectos fonético-fonológicos da língua foram observadas em tais investigações - ausência que chama a atenção, dado o importante papel desses aspectos na constituição dos sistemas alfabéticos de escrita.

Especialmente porque há, na literatura, relatos de investigações que tiveram como objetivo compreender aspectos mais minuciosos relacionados à ortografia no período da alfabetização, envolvidos na relação entre características ortográficas e aspectos fonético-fonológicos da língua. Nessas investigações, nota-se a preocupação em se compreenderem possíveis vínculos entre estrutura da sílaba e ortografia, já que a posição e/ou a complexidade silábica poderiam interferir no registro ortográfico convencional^(14-16)^. Notam-se, também, investigações que buscaram verificar a interferência da percepção e do treinamento auditivo na redução de erros ortográficos de base fonológica observados em crianças com desordem do processamento auditivo temporal^(17)^.

Encontram-se, além dessas, investigações baseadas em relações mais sistemáticas entre a fonologia e a ortografia, em que se observam tanto a análise de erros ortográficos como fonte reveladora do conhecimento de crianças acerca da fonologia da língua e de práticas de leitura e de escrita^(18)^_,_ quanto o desempenho ortográfico de crianças quanto ao registro de fonemas de classes fonológicas específicas^(19-22)^_._ Nestas últimas investigações, verificam-se indícios de como se dá o registro da relação fonema/grafema em função das classes fonológicas consonantais do PB. Verificam-se, também, indícios das possíveis zonas de conflito nessa relação. No entanto, não se observa, nesses trabalhos, preocupação com a diferença no estabelecimento da relação fonema/grafema no interior de uma mesma classe fonológica. A partir dessa lacuna, deriva-se a pergunta que norteou o desenvolvimento da presente investigação: em que medida o registro de grafemas que compõem uma mesma classe fonológica apresentaria pontos de simetria/não-simetria no registro ortográfico?

Para responder a essa pergunta, tem-se como hipótese que o registro ortográfico de fonemas de uma mesma classe seria influenciado pelo tipo de relação fonema/grafema que apresenta. Dessa forma, correspondências em que se observam relações de transparência seriam menos conflitantes e, portanto, menos suscetíveis a erros do que a ortografia de fonemas em que se observam relações de opacidade. Com base nessa hipótese, originaram-se os objetivos que orientaram o desenvolvimento da investigação relatada no presente artigo: (1) verificar em que medida a ocorrência de possíveis erros seria influenciada pelo tipo de relação (opaca/transparente) que os fonemas fricativos mantêm com os grafemas com que podem ser ortografados; (2) verificar, dentre esses dois tipos de relações, se os fonemas que apresentam relações grafêmicas comuns apresentam, ou não, diferenças entre si.

A escolha de se investigar a ortografia dos fonemas fricativos provém da complexidade observada na correspondência fonema/grafema observada nessa classe. Conforme exposto anteriormente, se considerarmos a posição de ataque silábico simples, embora predomine no PB a correspondência fonema/grafema motivada por relações transparentes - dos dezenove fonemas consonantais, doze apresentam correspondência regular nessa posição silábica^(23)^-, encontra-se nela também um número considerável de correspondências motivadas por relações opacas (sete, dos dezenove fonemas consonantais). O maior número das relações de opacidade nessas correspondências está justamente na classe dos fonemas fricativos, uma vez que quatro (/s/, /z/, /ʃ/, /ʒ/) dos seis fonemas que integram essa classe no PB apresentam escrita opaca, ou seja, irregular.

## MÉTODO

Para o desenvolvimento do presente artigo foi utilizado um conjunto de produções textuais retiradas de um banco de dados que integra o projeto *Vínculos entre características de práticas orais e letradas na aquisição e desenvolvimento da escrita* (CNPq - Processo 400183/2009-9), aprovado pelo Comitê de Ética em Pesquisa da FFC/UNESP sob o número 0138/2010.

O banco é composto por 2.972 produções textuais de 300 crianças que cursaram o Ensino Fundamental I, entre os anos de 2001 e 2004, em duas Escolas Municipais de Ensino Fundamental de São José do Rio Preto, estado de São Paulo. Compuseram o banco somente os textos das crianças autorizadas pelos pais ou responsáveis a participarem da tarefa mediante a assinatura do Termo de Consentimento Livre e Esclarecido^^*^^ .

As produções textuais foram coletadas por pesquisadoras do Grupo de Pesquisa ‘Estudos sobre a Linguagem’ (GPEL/CNPq). Aproximadamente a cada quinze dias, essas pesquisadoras realizavam oficinas pedagógicas que resultavam em diferentes propostas temáticas de escrita. Então, de acordo com o tema apresentado, as crianças deveriam produzir um texto. Essas propostas mobilizaram a produção de textos de diferentes gêneros discursivos por parte das crianças. Ressalte-se que as propostas obedeciam ao planejamento pedagógico das escolas. Desse modo, não se trata da configuração de um banco de dados com caráter experimental, mas, sim, com caráter mais próximo do cotidiano escolar das crianças.

Para a investigação relatada no presente artigo, do material do banco, foram selecionadas as produções textuais de todas as crianças que cursavam a 1ª série do Ensino Fundamental no ano de 2001 nas duas escolas, a saber: 75 crianças. Nenhuma delas apresentava qualquer queixa de aprendizagem da escrita nem, tampouco, de desenvolvimento de linguagem em sua fala. Trata-se, em síntese, de crianças com desenvolvimento típico tanto de fala quanto de escrita. Tais informações foram coletadas com seus pais e equipe pedagógica das escolas.

As produções são referentes a 14 propostas temáticas apresentadas ao longo do ano letivo. No Quadro 1 observam-se os temas de cada uma dessas propostas, bem como a data de apresentação e de coleta.

**Quadro 1:** Propostas temáticas

**Proposta**	**Tema**
1	Conhecimentos prévios sobre a audição
2	Relato da palestra sobre audição
3	Carta para a Renata 01
4	Carta para a Renata 02
5	O rato do campo e o rato da cidade
6	A verdadeira história dos três porquinhos: diário de um lobo
7	Precisando de óculos?
8	Dengue
9	Lista de compras 01
10	Lista de compras 02
11	Receita de bolo
12	Levantamento prévio sobre a voz
13	Palestra sobre a voz
14	Cartão de Natal

Fonte: Banco de dados da pesquisa.

Dessa forma, esperava-se, para análise, um total de 1.050 produções textuais (75 crianças x 14 propostas temáticas). No entanto, devido a ausências e à impossibilidade de interpretação de alguns registros, 750 produções foram analisadas e 300 produções foram descartadas.

Nas 750 produções analisadas, foram verificadas todas as ocorrências de grafemas que remetiam a fonemas fricativos do Português Brasileiro (PB), na posição silábica de ataque simples. Essas ocorrências foram classificadas em: ACERTOS, quando a grafia estava de acordo com as convenções ortográficas; e ERROS, quando a grafia estava em desacordo com a ortografia convencional. Tanto os acertos, quanto os erros, foram contabilizados em todos os fonemas fricativos alvo, /f/, /v/, /s/, /z/, /ʃ/ e /ʒ/, para se levantar a frequência de aparecimento de cada um desses fonemas na amostra.

Em outras palavras, para cada fonema alvo, primeiramente foi levantado o seu total de possibilidades de ocorrências em cada texto de toda a amostra analisada. Desse total, foi verificada a quantidade numérica de acertos e de erros. Em seguida, ainda para cada fonema alvo, essa quantidade foi transformada em números percentuais e em números relativos. Por exemplo: o fonema /f/ teve um total de 1604 possibilidades de registro em toda a amostra. Dessas 1604 possibilidades: 1562 corresponderam a acertos; 52 a erros. Em termos percentuais: 1604 possibilidades = 100%; 1562 acertos = 97%; 52 erros = 3%. Para se chegar aos números relativos, foi considerado o percentual de acertos (97%) e de erros (3%) divididos por cem. Chegou-se assim, respectivamente, aos números relativos de 0,97 e de 0,03. Considerando que os objetivos da investigação aqui em relato dizem respeito, prioritariamente, aos erros ortográficos, na análise estatística foram utilizados apenas os números relativos desses erros.

Tal procedimento foi necessário uma vez que a frequência de aparecimento de cada um desses fonemas, em uma amostra não-controlada, não ocorre de maneira semelhante. Por isso, a necessidade de se utilizar um número relativo para os erros, ou seja, um valor que variasse de 0 a 1 para efeito de análise estatística.

Os erros, por sua vez, foram agrupados (segundo o tipo de relação fonema/grafema) em: *erros em relações transparentes* /f/, /v/; e, em *erros em relações opacas* /s/, /z/, /ʃ/, /ʒ/. Tal agrupamento foi feito para que, de acordo com o primeiro objetivo, pudesse ser verificado se a ocorrência total de erros seria influenciada pelo tipo de relação (transparente/opaca) que os fonemas mantêm com os grafemas. Ainda, de acordo com o segundo objetivo, o agrupamento foi realizado de forma a verificar, dentre esses dois tipos de relações, se os fonemas que apresentam relações grafêmicas comuns apresentavam, ou não, diferenças entre si.

Os dados receberam tratamento estatístico com o uso do *software STATISTICA*, em sua versão 7.0. Nesse tratamento, foram realizadas análises descritiva e inferencial. Para a análise descritiva, foi utilizada uma medida de tendência central (média) e uma medida de dispersão (desvio padrão). Para a análise inferencial, foi estabelecido o nível de significância de α ≤ 0,05 e intervalo de confiança de 95%. Foram aplicados os testes não paramétricos: *ANOVA and Kendall Coefficient of Concordance* para análise de múltiplas variáveis dependentes simples; e o teste *Wilcoxon Matched and Pairs* para comparação entre duas variáveis simples e, também, para análise *post-hoc.* Em todas as análises, foram utilizados os valores correspondentes às medidas relativas.

## RESULTADOS

Buscou-se entender, como propõe o primeiro objetivo que orientou a investigação, em que medida os erros dependeriam das relações de transparência ou de opacidade que os fonemas fricativos mantêm com os grafemas com que podem ser ortografados. Para tanto, as 1.491 ocorrências de registros classificados como erros do total da amostra foram dispostas em dois grupos, cujos resultados estão expostos na Tabela 1:

**Tabela 1:** Comparação entre erros com base no tipo de relação fonema/grafema.

**Relação F/G**	**Nº de ocorrências**	**Média**	**Desvio Padrão**	**Análise inferencial**
Transparente	112	0,78	0,13	T = 3,0
				Z= 7,3
Opaca	1379	0,89	0,19	p = 0,00 (*)

Fonte: dados da pesquisa.

**Legenda:** F/G - Fonema/Grafema;

(*) indica diferença estatística. Teste: *Wilcoxon Matched Pairs.*

Como se pode observar na Tabela 1, 112 erros (7,51%) ocorreram em fonemas cuja ortografia é transparente e 1.379 (92,49%) naqueles de ortografia opaca, distribuição que se mostrou significativa em termos estatísticos.

Assim como proposto no segundo objetivo, foi verificado, dentre os dois tipos de relações (opaca/transparente), se os fonemas que apresentam relações grafêmicas comuns mostravam diferenças entre si. Os resultados referentes à comparação entre os fonemas que apresentam relação grafêmica transparente estão expostos na Tabela 2:

**Tabela 2:** Comparação entre erros ortográficos de fonemas com ortografia transparente

**Fonema alvo**	**Nº de ocorrências**	**Média**	**Desvio Padrão**	**Análise inferencial**
/f/	*52*	0,22	0,38	T = 263,50
				Z= 0,58
/v/	*60*	0,26	0,4	p = 0,56

Fonte: dados da pesquisa. Teste *Wilcoxon Matched Pairs*.

Quanto à comparação entre os fonemas com relação fonema/grafema transparente, exposta na Tabela 2, verifica-se que o número de ocorrências de erros nos fonemas /f/ e /v/ é bastante próximo, 52 (46,43%) e 60 (53,57%), respectivamente. Com valores de média e de desvio padrão também semelhantes. Na análise inferencial, não foi observada diferença estatística significativa na distribuição dos erros.

Ainda com relação ao segundo objetivo dessa investigação, na Tabela 3, estão expostos os resultados da comparação entre os fonemas que apresentam relação opaca com os grafemas pelos quais podem ser ortografados:

**Tabela 3:** Comparação entre erros ortográficos de fonemas com ortografia opaca

**Fonema alvo**	**Nº de ocorrências**	**Média**	**Desvio Padrão**	***Sum of ranks***	**Análise inferencial**
/s/	920	0,64	0,23	281^1,2,3^	Chi- sqr. 127,92
/z/	188	0,15	0,17	170^1,4^	df = 3
/ʃ/	193	0,13	0,13	169^2,5^	p= 0,00*
/ʒ/	78	0,05	0,07	120^3,4,5^	

Fonte: dados da pesquisa. Teste Friedman ANOVA and Kendall Coeff. of Concordance.

**Legenda:** (*) indica diferença estatística significativa. Análise post-hoc: teste Wilcoxon Matched Pairs, números sobrescritos iguais indicam diferença estatística significativa.

Quanto à comparação entre os fonemas com relação fonema/grafema opaca observa-se: (i) maior ocorrência de erros na grafia do fonema /s/ (920 - 66,71%); (ii) ocorrência em valores absolutos e percentuais próximos entre os fonemas /z/ e /ʃ/, na ordem, 188 (13,63%) e 193 (13,99%); e (iii) menor número de ocorrências na grafia do fonema /ʒ/, 78 (5,67%). Quanto à análise inferencial, foi observada diferença estatística significativa na comparação entre os fonemas dessa categoria. Na análise *post-hoc* verificou-se que o fonema /s/ se diferencia de todos os demais fonemas; os fonemas /v/ e /ʃ/, por sua vez, apresentaram diferenças apenas com os fonemas /s/ e /ʒ/; e, por fim, o fonema /ʒ/ apresentou diferenças com os fonemas /s/, /z/ e /ʃ/.

Finalizada a apresentação dos resultados, serão apresentadas hipóteses explicativas para as tendências para as quais eles apontam.

## DISCUSSÃO

No que se refere à tendência encontrada para o primeiro objetivo, em seu conjunto, a ocorrência de erros se mostrou como dependente do tipo de relação que os fonemas mantêm com os grafemas. Ou seja, observou-se que fonemas que apresentam relação transparente com os grafemas com que podem ser ortografados representaram uma porcentagem bastante pequena (7,51%) da ocorrência de erros quando comparados aos fonemas que apresentam relação grafêmica opaca. Esses últimos corresponderam à maior parte da amostra (92,49%).

A principal hipótese explicativa para essa tendência é a do número de possibilidades grafêmicas que os fonemas apresentam. Com efeito, nas relações de transparência, um fonema só apresenta uma possibilidade de registro; nas relações de opacidade, por sua vez, um fonema pode apresentar duas ou mais possibilidades de registro; logo, a probabilidade de ocorrência de erros aumenta substancialmente.

Verifica-se, portanto, nessa primeira tendência um importante aspecto a ser considerado na ocorrência de erros ortográficos: o tipo de relação que o fonema mantém com o grafema pelo qual pode ser ortografado. Embora voltados para a leitura e desenvolvidos com embasamento teórico divergente daquele do presente artigo, dois trabalhos observam indícios de possíveis zonas de facilidades de decodificação de leitura em correspondências fonema/grafema com ortografia transparente^(10,11)^. Também em trabalho desenvolvido com outro tipo de enquadramento teórico-metodológico e voltado para características globais da escrita (e não apenas as ortográficas), foram destacadas possíveis zonas de dificuldades em correspondências com ortografia opaca^(12)^.

Ainda com relação à primeira tendência, encontramos investigações também baseadas em aspectos fonético-fonológicos da língua que levantaram aspectos importantes que podem interferir na ocorrência de erros ortográficos, como: (i) as classes fonológicas^(21)^ e (ii) a complexidade silábica^(16)^.

Quanto às tendências encontradas para o segundo objetivo, primeiramente, não foi observada diferença entre os fonemas que apresentam ortografia transparente (/f/ e /v/) na ocorrência de erros. Esse resultado sugere que esses fonemas funcionam de modo muito semelhante na ortografia, possivelmente por apresentarem uma única possibilidade ortográfica, reduzindo, dessa forma, a probabilidade de serem ortografados com erros. Essa tendência confirma aquela verificada com dados de sistemas de escrita que apresentam alto nível de regularidades entre fonemas e grafemas, como o italiano, o finlandês e o indonésio, em que se observam fortes reflexos da fonologia na ortografia. Essas regularidades diminuem a possibilidade da ocorrência de erros em tarefas de leitura e de escrita quando comparada à ocorrência de erros em sistemas de escrita com alto grau de opacidade, como o inglês, por exemplo^(25)^.

A segunda tendência encontrada, também para o segundo objetivo, foi a não-simetria entre a ocorrência de erros na ortografia dos fonemas que apresentam relação opaca com seus grafemas correspondentes (/s/, /z/, /ʃ/ e /ʒ/). Dessa forma, apesar de manterem o mesmo tipo de relação, esses fonemas não demonstram funcionar de maneira semelhante na ortografia. Uma possível hipótese explicativa para essa tendência é a presença das múltiplas possibilidades de grafia que um mesmo fonema admite. Nos fonemas fricativos, seu registro ortográfico pode apresentar de duas a nove possibilidades grafêmicas na posição silábica de ataque simples. Além desse aspecto quantitativo, dentre essas possibilidades, podem operar: (a) regras de posição silábica - por exemplo, quando registrado entre vogais, o grafema {s} remete ao fonema /z/; esse mesmo grafema, quando registrado em início de palavra (**s**anta), ou após grafemas que remetem a fonemas da classe das líquidas (aniver**s**ário) e das nasais (man**s**ão), passa a remeter ao fonema /s/; (b) ocorrência de dígrafos - situação em que o fonema apresenta como possibilidade grafêmica a combinação de dois elementos, por exemplo, as letras ‘c’ e ‘h’, quando juntas ‘ch’ passam a remeter ao fonema /ʃ/; e (c) relações de concorrência - quando dois ou mais grafemas podem ser utilizados na mesma posição e no mesmo contexto silábico (“música”; “zebra”; “exame”, situação em que todos os grafemas remetem ao fonema /z/)^(1)^.

Considerando, ainda, a não-simetria entre os fonemas fricativos que apresentam ortografia opaca, foram observadas ainda: (i) maior ocorrência de erros na grafia do fonema /s/; (ii) comportamento semelhante entre os erros dos fonemas /z/ e /ʃ/; (iii) não-relação entre os erros ocorridos na grafia do fonema /ʒ/ com os demais fonemas fricativos. Essas tendências sugerem que, além de apresentarem divergências, apresentam, ainda, uma gradação.

Nesse sentido, a maior ocorrência de erros na grafia do fonema /s/ pode ser explicada pela grande opacidade que mantêm, uma vez que, na posição silábica de ataque simples, esse fonema apresenta nove possibilidades de ser ortografado {s, ss, c, ç, sc, sç, xs, xc, x}, sendo o fonema mais opaco quando comparado a todos os demais do PB (tanto da classe das fricativas, quanto das demais classes: oclusivas, nasais, líquidas e vogais). Além desse grande número de possibilidades ortográficas, observam-se outras complexidades em sua ortografia como: (a) possibilidade de dígrafos - por exemplo, a escrita das palavras {a**ss**ar} {e**xc**eção}; {n**as**cer}; (b) relações de concorrência - como nas palavras {**s**ela}, {**c**eia}, {pa**ss**eio}, {a**c**eite} em que todos os grafemas remetem ao fonema /s/; e (c) número reduzido de regras contextuais que prevêem o uso de um ou outro grafema, sendo necessário, na maioria das vezes, a memorização. Assim, grafar esse fonema de acordo com as convenções ortográficas pode ser uma tarefa muito difícil para crianças de séries iniciais. Resultado similar foi observado na análise de erros ortográficos nas séries iniciais, em que a grafia envolvendo o fonema /s/ correspondeu à maior parte dos erros encontrados^(18)^.

O comportamento semelhante observado na ocorrência de erros na grafia dos fonemas /z/ e /ʃ/, por sua vez, pode estar associado a duas características da ortografia desses fonemas: número reduzido de possibilidades grafêmicas e apenas uma possibilidade de dígrafo. O registro ortográfico do fonema /z/ admite três possibilidades grafêmicas {z}, {s}, {x}, sem nenhuma possibilidade de dígrafo; já o registro ortográfico do fonema /ʃ/, embora admita somente duas possibilidades de registro, {x}, {ch}, uma delas se dá por meio de dígrafo. Ainda, nenhum desses dois fonemas apresenta regra que determine o uso de seus grafemas de acordo com a ortografia, já que “quando mais de uma letra pode, na mesma posição, representar o mesmo som, a opção pela letra correta em uma palavra é, em termos puramente fonológicos, inteiramente arbitrária.”^(1:21)^.

Na terceira tendência encontrada dentre os erros que envolviam relação fonema/grafema opaca, verificou-se que o fonema /ʒ/ não apresentou relação com nenhum dos outros três fonemas com escrita opaca /s/, /z/, /ʃ/. Quando comparado aos demais fonemas fricativos, o fonema /ʒ/ apresenta apenas duas possibilidades de registro gráfico {j} e {g}. Dentre essas duas possibilidades, uma pode ser prevista por regras contextuais, uma vez que o grafema {g} só corresponde ao fonema /ʒ/ quando precedido das vogais /e/ e /i/. Além disso, a grafia desse fonema não admite a possibilidade de dígrafos, reduzindo, ainda mais, a complexidade para ortografá-lo. Dessa forma, considerando esse resultado, pode-se inferir que esse fonema se comporta ortograficamente numa gradação intermediária entre os fonemas com escrita transparente e os fonemas com escrita opaca.

Considerando todas as tendências encontradas quanto às características da relação entre aspectos fonético-fonológicos e aspectos ortográficos dos fonemas fricativos, verificam-se indícios de que, embora esses fonemas funcionem de maneira semelhante na fala, o mesmo não ocorre no aspecto ortográfico da escrita. Na fala, esses fonemas costumam funcionar em pares que se diferenciam por uma unidade mínima que, nesse caso, é a presença ou ausência da característica do traço de vozeamento. Assim, na fala de crianças com desenvolvimento fonológico típico e atípico podem ocorrer estratégias de reparo que envolve a dessonorização - por exemplo, o fonema /v/ ao ser produzido sem a vibração das pregas vocais corresponde ao fonema /f/^(26)^. Porém, nos resultados encontrados, verificou-se que, na ortografia, outros fatores podem influenciar o registro desses fonemas, como o número de possibilidades grafêmicas, a ocorrência de dígrafos e a presença/ausência de regras que definem o uso de um grafema de acordo com a norma ortográfica vigente.

Por fim, ainda em relação às tendências encontradas, embora o PB apresente um sistema de escrita predominantemente transparente e, de acordo com a literatura, sistemas com escrita regular estejam menos suscetíveis a equívocos por parte das crianças em alfabetização^(25)^, observa-se a importância de analisar especificamente os fonemas que compõem uma mesma classe fonológica. Além disso, observa-se a importância de caracterizar como se dá o desenvolvimento das relações que as crianças consideradas típicas estabelecem entre fonemas e grafemas e quais são suas possíveis zonas de conflito, uma vez que, na literatura a ocorrência de erros de leitura e de escrita, sobretudo em seu aspecto ortográfico, são utilizados como critérios de diagnóstico de transtornos de aprendizagem^(6,7,25)^.

Os resultados encontrados nessa pesquisa podem contribuir para uma melhor compreensão sobre as possíveis facilidades e/ou dificuldades ortográficas nas séries iniciais da alfabetização, por parte dos profissionais dos campos da saúde e da educação. Especialmente porque os resultados apontam para a importância de esses profissionais terem conhecimento sobre as relações, às vezes bastante complexas, entre aspectos fonético-fonológicos da língua e aspectos das convenções que regulam a ortografia, para que esse conhecimento forneça subsídios para as práticas clínicas e educacionais com a escrita infantil.

Os resultados e tendências encontrados podem direcionar novas investigações que visem caracterizar a natureza dos erros ortográficos em diferentes classes fonológicas, em outros contextos silábicos e, ainda, na sequência dos anos que compõem o Ciclo de Alfabetização. Expõem-se, assim, as limitações do estudo aqui apresentado.

## CONCLUSÃO

Os resultados encontrados na presente pesquisa permitiram verificar que, em seu conjunto, os fonemas em que se observam relações de transparência com seus grafemas correspondentes foram muito menos suscetíveis à ocorrência de erros do que os fonemas que mantêm relações opacas com seus grafemas correspondentes. Esses resultados corroboram, parcialmente, a hipótese inicial de que o registro ortográfico de uma mesma classe seria influenciado pelo tipo de relação fonema/grafema que apresenta.

Entretanto, diferentemente do que se previa na hipótese, dentre os opacos, fatores como: (a) o número de possibilidades grafêmicas; (b) concorrência entre grafemas, ou seja, ausência ou número limitado de regras que definem o uso ortograficamente adequado; e (c) presença/ausência de dígrafos, interferiram na ocorrência de erros.

Foi possível verificar pontos de simetria entre fonemas com ortografia transparente, uma vez que os erros ocorreram de maneira semelhante e em menor número; e pontos de não-simetria dentre os fonemas que apresentam ortografia opaca. Dadas essa simetria e essa assimetria nos erros, os resultados obtidos apontam para uma gradação em sua ocorrência, que varia em função da transparência e do grau de opacidade nas relações entre fonemas e grafemas de uma mesma classe. Essa gradação pode ser dividida em quatro grupos, considerando-se a seguinte ordem das relações mais transparentes às mais opacas: 1º /f/ e /v/; 2º /ʒ/; 3º /z/ e /ʃ/; e 4º /s/.

## Footnotes

* Embora, de acordo com a resolução do Conselho Nacional de Saúde – CNS de Nº 466/12^24^ , seja obrigatória a autorização tanto dos pais quanto das crianças participantes, ressaltamos que a coleta ocorreu em período anterior a essa resolução, em que somente o assentimento dos pais e/ou responsáveis permitia a coleta de dados.
